# Case report: Robotically-treated spontaneous interstitial pregnancy on tubal stump

**DOI:** 10.3389/fmed.2024.1473307

**Published:** 2024-11-20

**Authors:** Mario Ascione, Luigi Della Corte, Giuseppe D’Angelo, Mario Palumbo, Rafał Watrowski, Attilio Di Spiezio Sardo, Giuseppe Bifulco

**Affiliations:** ^1^Department of Public Health, University of Naples Federico II, Naples, Italy; ^2^Department of Neuroscience, Reproductive Sciences, and Dentistry, School of Medicine, University of Naples Federico II, Naples, Italy; ^3^Department of Obstetrics and Gynecology, Helios Hospital Müllheim, Müllheim, Germany; ^4^Faculty of Medicine, University of Freiburg, Freiburg, Germany

**Keywords:** interstitial pregnancy, ectopic pregnancy, robotic surgery, fertility, case report

## Abstract

To report a rare case of a right interstitial pregnancy spontaneously occurring in a patient who had previously undergone homolateral salpingo-oophorectomy, and to propose possible explanations for the mechanisms involved in the genesis of this rare scenario. A 32-year-old G3P1 female presented to our emergency room with symptoms related to a suspected ectopic interstitial pregnancy managed in another hospital using a conservative pharmacological approach. After discussing the risks, firstly she underwent a transvaginal ultrasound examination, then a diagnostic hysteroscopy to clarify the unclear ultrasound finding, followed by a successful robot-assisted laparoscopic cornual resection. Hysteroscopy demonstrated an empty uterine cavity, confirming the suspect of pregnancy localization into the interstitial portion of the tubal stump. Through the robot-assisted laparoscopic approach, all the trophoblastic tissue was removed without causing significant damage to the surrounding myometrium and preserving the patient’s fertility. No post-operative complications were recorded. The robotic approach successfully allowed the cornual resection, with minimal blood loss and optimal suturing of the uterine defect. Although our knowledge is still limited, it is possible that the pregnancy nested in the tubal residue after being properly fertilized into the intact tube. However, it cannot be ruled out that there have been remodeling phenomena of the tubal residue so that it has acquired the ability to intercept the oocyte.

## 1 Introduction

Ectopic pregnancy is defined as a blastocyst implantation outside the uterine cavity with a fallopian tube placement rate of ≥95.5%. Interstitial pregnancies (IPs) represent 2–4% of ectopic pregnancies (1). The “interstitial pregnancy” is defined as the gestational sac implant within the proximal tubal segment, which is located within the uterine wall muscles (2). A correct diagnosis of IP may be difficult, and it necessitates appropriate ultrasound interpretation and training. Treatment options include conservative medical management or surgical intervention.

## 2 Case description

**Figure 1:**
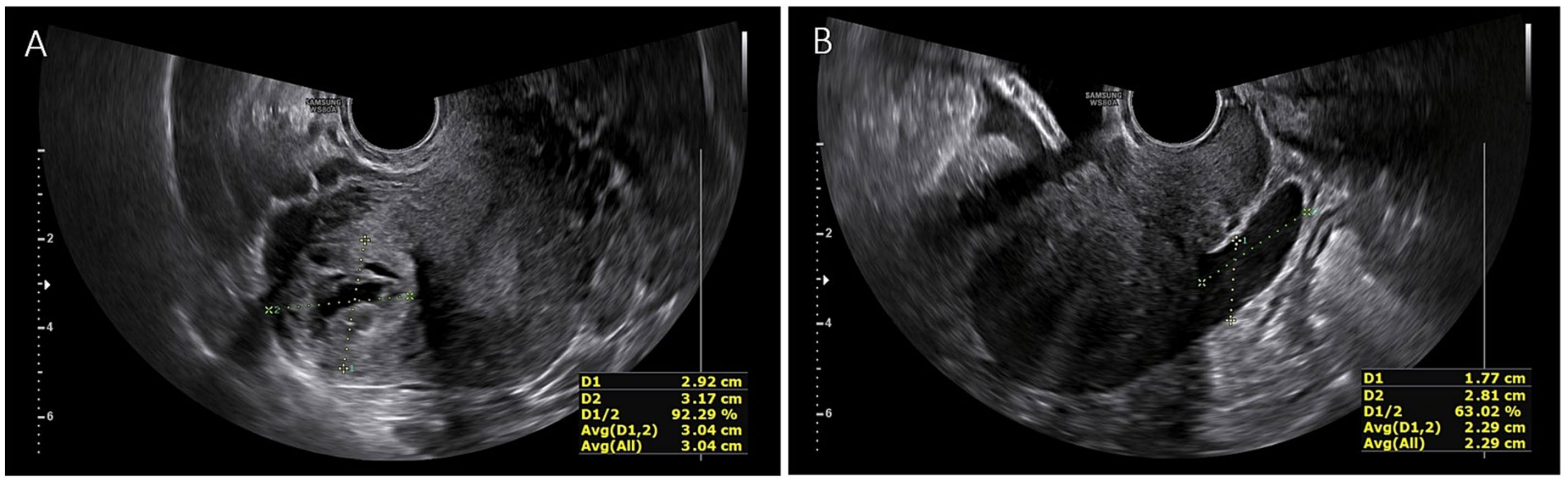
**(A)** Ultrasonographic finding. A 25×26 mm neoformation, with an intense peripheral vascularization in the right angular area suggesting an ectopic pregnancy; **(B)** Free fluid in the pouch of Douglas.

**Figure 2:**
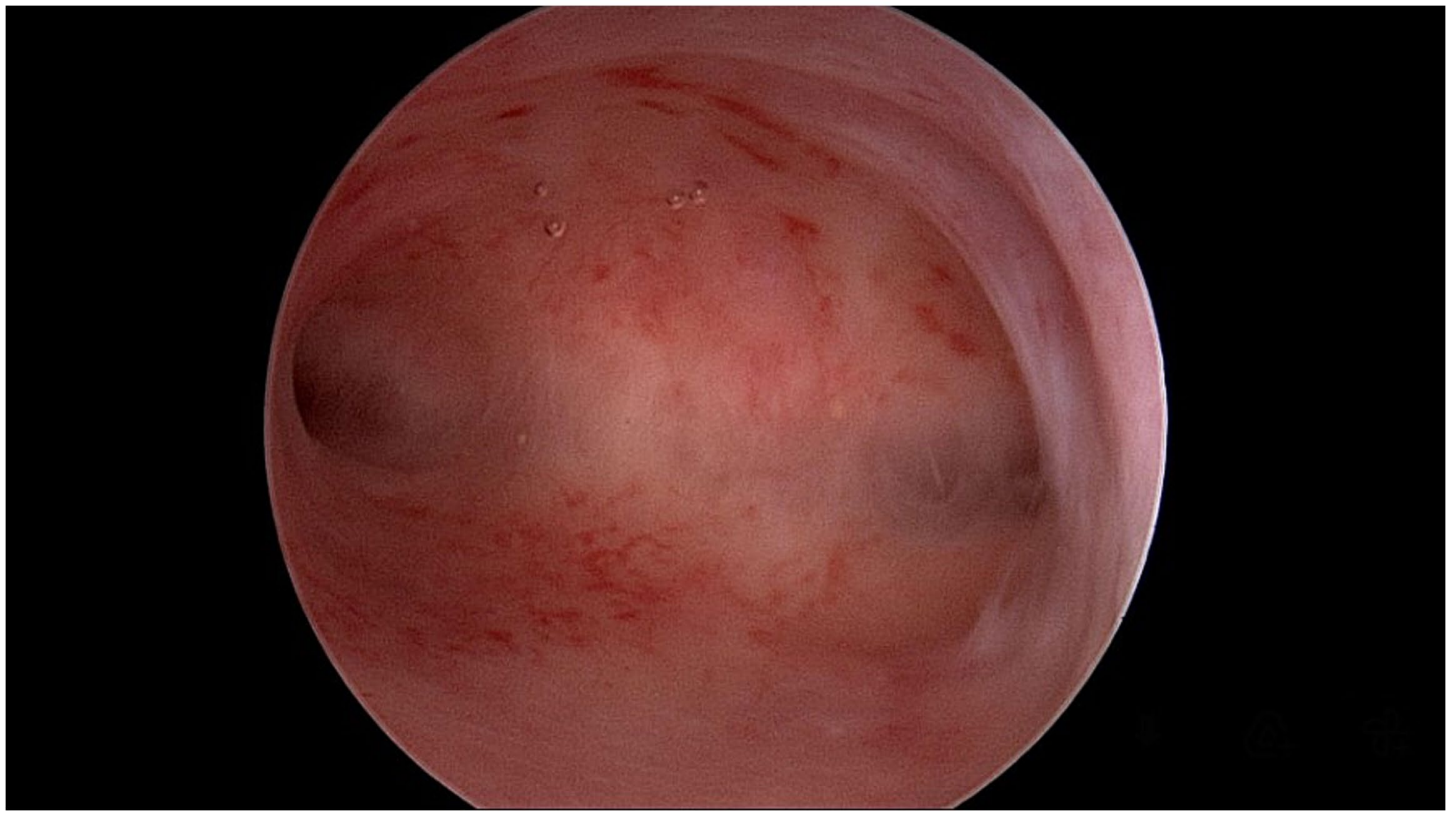
Diagnostic hysteroscopy shows an empty uterine cavity with visible tubal ostia.

**Figure 3:**
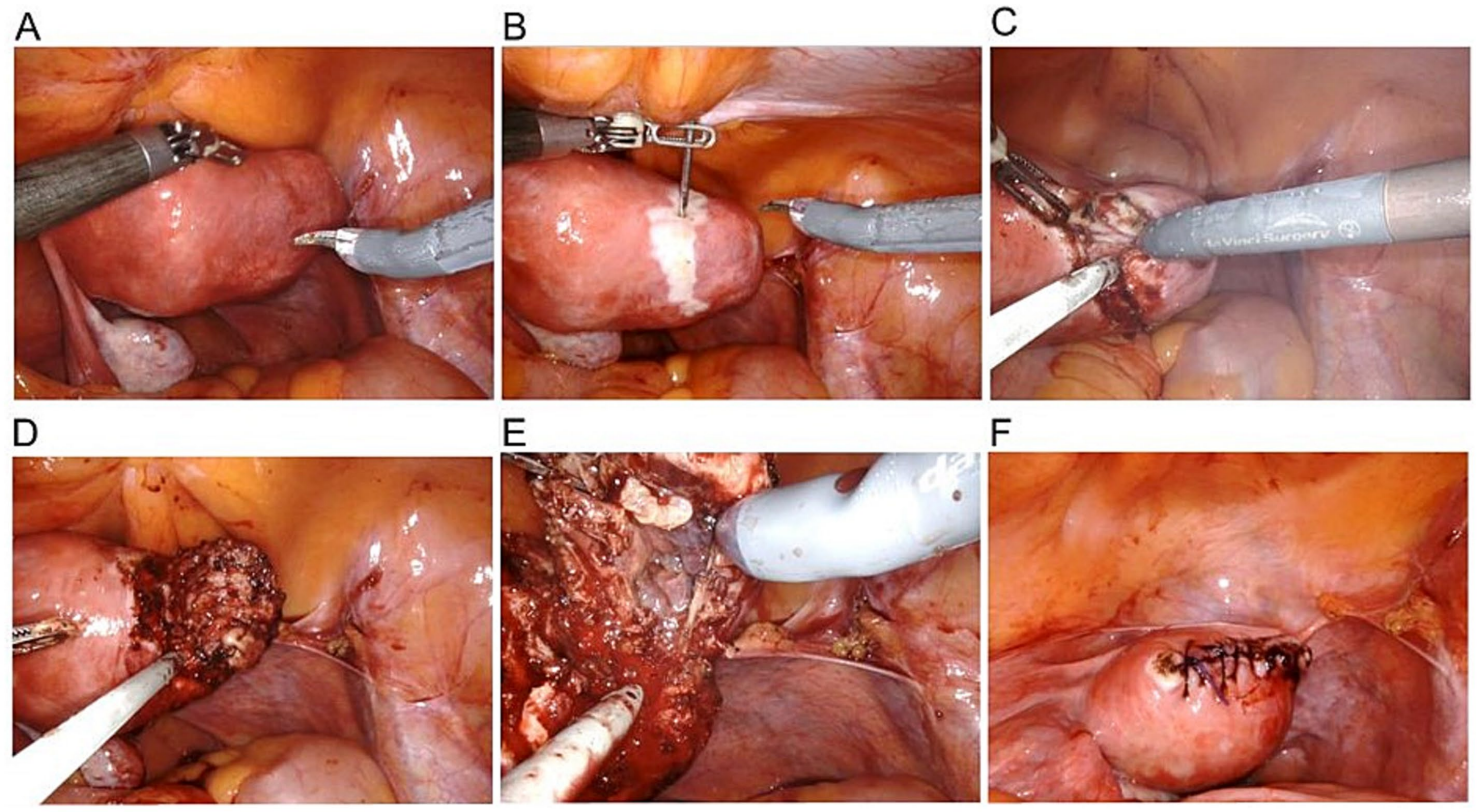
**(A)** Robotic view of interstitial pregnancy; **(B)** Perilesional injection of 20 U of diluted vasopressin in 100 mL of normal saline solution; **(C)** Incision of the serosa and cleavage plan identification; **(D)** Pregnancy and surrounding myometrium resection; **(E)** Gestational sac; **(F)** Uterine wall repair with double-layer suture.

A 32-year-old woman, G3P1, presented to our emergency room complaining of lower abdominal pain (Visual Analogue Scale (VAS) score: 8) with no vaginal bleeding. The patient had a surgical history of a right laparotomic adnexectomy when she was a child because of a cystic teratoma. According to her health records, the woman was attempting to conceive, but in April 2023, a different hospital admitted her as a suspect of cornual pregnancy with a serum beta human chorionic gonadotropin (*β*-hCG) level of 15,000 mUI/mL and an ultrasound showing the presence of a gestational sac with a single live embryo, with a crown-rump length (CRL) of 6 mm, and biometry corresponding to 6 weeks +2 days located in the right angular area of the uterus. The patient was first treated with methotrexate 50 mg twice, one week apart. The first *β*-hCG level determination was made 48 h later, and then every 24 h showed a decrease (T1: 8,782; T2: 7,882; T3: 7,261; T4: 6,152 mlU/ml) until June 2023, when she arrived at our emergency room. She had fair condition: her vital signs were stable and within normal limits. She was conscious, though she felt uncomfortable. The patient reported spontaneous pelvic pain which was difficult to localize. At the abdominal physical exam, tenderness to deep palpation was noted in her right lower quadrant radiating to the hypogastrium. Blumberg and Rovsing signs were negative. An ultrasound examination was performed, revealing in the right angular area the presence of a 25×26 mm neoformation, with an intense peripheral vascularization suggesting an ectopic pregnancy in the right angular area and little free blood in the pouch of Douglas (Figure 1). The blood tests showed: hemoglobin 10.4 g/dL; white blood cell (WBC) count 7,800/mL, with 70% neutrophils; C-reactive protein (CRP) and Erythrocyte Sedimentation Rate (ESR) were just slightly increased; serum *β*-hCG level 25.4 mUI/mL. The patient was admitted to our Obstetrics and Gynecology Unit, and a diagnostic hysteroscopy was performed, which showed a regular endometrial cavity, regular tubal ostia, and no neoformation inside the uterine cavity (Figure 2). So, a diagnosis of interstitial ectopic pregnancy on the tubal stump was made. Although *β*-hCG levels were declining and a wait-and-see approach would have been appropriate, due to the persistence of symptoms, the ultrasound evidence of a richly vascularized formation, the reproductive desire, and the peculiar pathological condition to be addressed, we opted for the surgical approach after detailed counseling and obtaining written informed consent. Furthermore, in our experience with ectopic pregnancies (3), the choice of methotrexate in this case was questionable from the outset, and surgery seemed the preferred route. A robotic-assisted laparoscopy was performed using the *Da Vinci® Robotic Surgical System*. At the introduction of the optical trocar, the presence of oval tumefaction corresponding to the right corner of the uterine was observed (Figure 3A). This finding was compatible with the suspicion of ectopic pregnancy, of course. The left ovary appeared normal. The right ovary was absent for prior surgery. It was not possible to distinguish the boundary between the uterus and the remaining tubal portion due to the alteration of the usual anatomical relationships. No hemoperitoneum in the pouch of Douglas was detected. After the injection of a vasoconstrictor agent inside the pregnancy (20 U of diluted vasopressin in 100 mL of normal saline solution), an incision of the serosa was made (Figures 3B–C). The dissection plane between the myometrium and the suspected gestational sac was identified. So, without damaging the endometrial cavity, the pregnancy as well as surrounding tissue were removed, and an accurate hemostasis on the uterine breach was obtained. Hence, a wedge resection of the right angular part of the uterus (Figures 3D–E). The tissue samples were placed in an endo-bag and extracted through one of the laparoscopic accesses. The uterine wall was then repaired with a double-layer suture by self-blocking monofilament (*V-Loc 2.0* barbed-suture type) (Figure 3F). Eventually, the samples were analyzed by pathologists. The histology confirmed the diagnosis, showing the presence of decidual cells and fragments of myometrium with adenomyosis. The procedure had no complications. The patient was discharged 48 h after surgery. The clinical conditions were satisfactory, and the patient had an immediate return to daily activities without complaining of any symptoms.

## 3 Discussion

**Figure 4:**
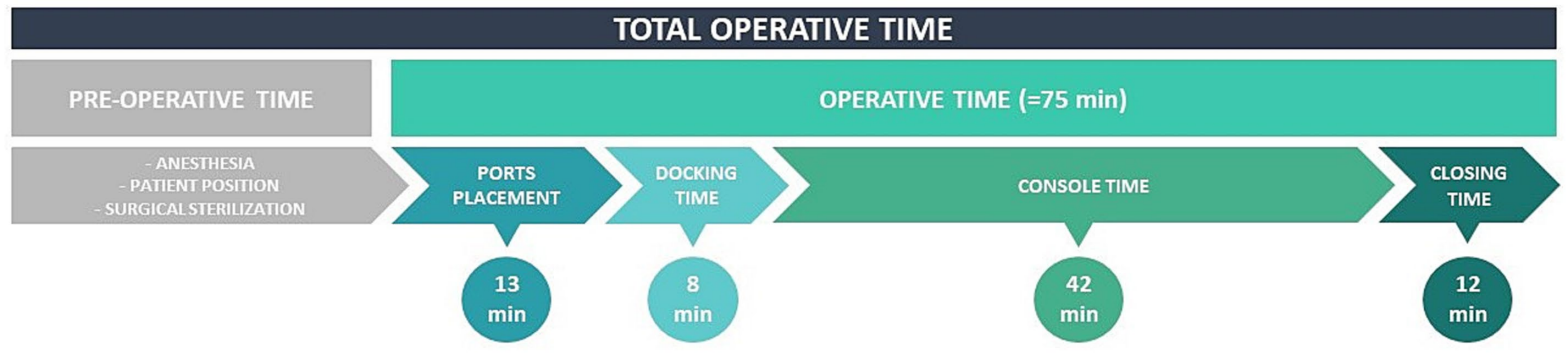
Summary of operating room timeline.

The most common site of ectopic pregnancies is the fallopian tube. Only 2 to 4% of all ectopic pregnancies occur in the interstitial or cornual part of the uterus. The terms cornual and interstitial ectopic pregnancy have been used interchangeably until now. According to literature, the rudimentary horn of a unicornuate uterus or of a septate/bicornuate uterus is where the true cornual pregnancy occurs (4, 5). The angular pregnancy is also frequently mistaken for an interstitial or cornual pregnancy. Angular pregnancies develop anatomically just medial to the utero-tubal junction, at the lateral angle of the endometrial/uterine cavity, and medial to the round ligament (5). Since there is not agreement on the exact ultrasonic characteristics of these three entities, the literature improperly interchanges them. While interstitial or cornual pregnancies can be considered ectopic pregnancies to be terminated, an angular pregnancy should be regarded as a possibly viable intra-uterine eccentric pregnancy since it may be carried to term in some cases (6). Patients may complain of vaginal bleeding or abdominal pain, be asymptomatic, or have their IP discovered after an ordinary early pregnancy ultrasound. Only patients with a diagnosed IP who are hemodynamically stable and have no clear concerns of early rupture, such as large gestational sac or rapidly rising *β*-hCG levels, should be considered for conservative therapy (both expectant and medical management) (7). For women with an IP with declining serum β-hCG levels (regardless of ectopic mass size or baseline serum β-hCG levels), expectant care is an acceptable first-line strategy (8, 9). Single-dose or multiple-dose courses of methotrexate are employed in medical management. With a failure rate for conservative medical care ranging from 9 to 65% in prior studies, surgical intervention may still be required if the ectopic pregnancy ruptures. A feasible and safe alternative to systemic methotrexate administration is direct methotrexate injection into the interstitial ectopic pregnancy. Historically, the likelihood of the effectiveness of conservative treatment was estimated using a *β*-hCG threshold of 5,000.00 mIU/mL. Surgical management of IPs represents an essential option since it provides permanent treatment. Women with IPs who are hemodynamically unstable and/or have ultrasound findings suggesting an incipient or recent pregnancy rupture should have prompt surgery. Patients who receive expectant or medical treatment are at a higher risk of persistent interstitial pregnancy and must be monitored for serial *β*-hCG values until resolution. The laparoscopic treatment of interstitial pregnancies has been becoming more frequently performed (8), supplanting the classic laparotomic approach. Laparoscopic treatment provides some advantages over laparotomy, including a shorter hospital stay, a faster return to normal activities, and fewer healthcare expenses (10). Over the last few decades, many different kinds of techniques have been developed, including cornuostomia, salpingotomy, and cornual resection (11). Regardless of the surgical technique, blood loss is an inherent hazard of the surgical program. Due to the extremely vascularized interstitial pregnancies (12), multiple strategies can be used before making a cornual incision to minimize intraoperative blood loss: vasopressin injection into the peri-cornual area, electric cauterization of the incision area, endo-loop application to create a para-cornual tourniquet, and an encircling suture around the cornua. Worries are related to electrocoagulation procedure which would weaken the area and possibly increase the risk of uterine rupture in the future by harming the myometrium underneath and delaying the revascularization process. We believe that cautious coagulation of the surrounding myometrium does not compromise the uterine integrity but rather helps to avoid post-operative bleeding. In addition, we maintain that the use of intralesional vasoconstrictors is essential to decrease the hemorrhagic risk and guarantee the surgeon a clean surgical field. To date, the use of robotic surgery for the management of ectopic pregnancies has already been described in the international literature. Ansari et al. reported the first description of robot-assisted cornual ectopic excision, listing the advantages of this technique (13). Robot-assisted surgery (RAS) has been criticized for longer operative times compared to traditional laparoscopy. Some procedures, particularly difficult ones, may actually take less time to complete due to the increased precision provided by the robotic tools and wider eyesight (14, 15). Indeed, in our experience, three-dimensional and magnified vision enables greater overall accuracy, the breadth of surgical gestures simplifies difficult maneuvers, such as suturing, and significantly reduces operating times. In addition to the well-known benefits of laparoscopy, RAS allows for precision surgery. This results in greater respect for anatomy and minimal healthy tissue injury, which we hypothesize may play a role in preserving fertility. About the “docking time” that affects the *Da Vinci® Robotic Surgical System*, the most important variable is surely the experience of the operating team. Indeed, many studies have analyzed robotic surgical learning curves on the *Da Vinci* platform and have suggested that the longer operative times associated with RAS decrease as surgeons become more familiar with the technology (16). Through adequate training, our staff has acquired the right skills to perform robotic docking and set-up time-effectively, so the overall operating time was not much different from that of conventional surgery (Figure 4). Previous tubal surgery constitutes an independent risk factor for the development of ectopic pregnancies (17). Considering that our patient underwent monolateral salpingo-oophorectomy during childhood, it remains to be explained how the pregnancy was implanted in the interstitial tubal portion. To hypothesize about the circumstances leading to this scenario, it is plausible that the oocyte has been fertilized in the intact tube and subsequently migrated into the control-lateral tubal stump. This transfer could be facilitated by intrauterine fluid currents. Alternatively, some Authors suggest that an ovum could have transmigrated and passed through a fistula into the tubal stump, where successive sperm fertilization and local embryo nidation occurred (18). However, unknown is the mechanism that could allow the oocyte to be intercepted by a mutilated tube, in which the fimbrial structures that should capture it are now abolished. An attractive explanation might be that the tubal stump remodeled throughout the years, gaining the ability to intercept the oocyte released by the contralateral ovary. Moreover, to support this proposal, there is some evidence that the uterus undergoes remodeling processes after surgical procedures (19). So, considering the possibility of future remodeling of the uterus, it is reasonable to assume the precision of the robotically assisted system was perfectly suited to this rare condition. The only studies found in the literature referring to ectopic pregnancy after a previous tubal surgery concern patients with prior salpingectomy undergoing *in-vitro* fertilization (IVF) and embryo transfer (20). The strength of this work is that we have described an absolutely rare case with truly innovative surgical management. The hope is to encourage scientific research on this topic, considering that the literature is still rather limited, and to stimulate collective interest in deepening the etiopathogenesis of ectopic pregnancies. However, the superiority of robotic surgery over traditional laparoscopy remains to be defined in a larger case study. Indeed, the main limitation of our work is that a single case of interstitial pregnancy on the tubal stump is described, which limits generalizability. Unfortunately, interstitial pregnancies are very rare. As a result, it is difficult to understand if our approach is applicable on a large scale.

## 4 Conclusion

IP remains a truly rare condition. We believe that robotic surgery represents a feasible and safe strategy for the surgical treatment of IPs and can offer some advantages, such as shorter surgical time, magnification of the operative field, wide mobility of the robotic arms, minimal invasiveness, and minimal blood loss, while minimizing the risks. Nevertheless, a pilot study could validate our positive surgical management. Finally, further evidence is needed to conclusively explain the pathophysiological mechanisms underlying the development of spontaneous IPs on the tubal stump.

## Data Availability

The original contributions presented in the study are included in the article/supplementary material, further inquiries can be directed to the corresponding author/s.
